# Characterizing cognitive aging of associative memory in animal models

**DOI:** 10.3389/fnagi.2012.00010

**Published:** 2012-09-12

**Authors:** James R. Engle, Carol A. Barnes

**Affiliations:** ^1^Evelyn F. McKnight Brain Institute and ARL Division of Neural Systems, Memory and Aging, University of ArizonaTucson, AZ, USA; ^2^California National Primate Research CenterDavis, CA, USA

**Keywords:** associative learning, delay conditioning, trace conditioning, optimization, cognitive assessment battery

## Abstract

An overview is provided of the simple single-cue delay and trace eyeblink conditioning paradigms as techniques to assess associative learning and memory in the aged. We highlight and focus this review on the optimization of the parameter space of eyeblink conditioning designs in the aged to avoid and control for potential confounds that may arise when studying aged mammals. The need to examine the contribution of non-associative factors that can contribute to performance outcomes is emphasized, and how age-related changes in the central nervous system as well as peripheral sensory factors can potentially bias the interpretation of the data in the aged is discussed. The way in which slight alterations of the parameter space in the delay and trace eyeblink conditioning paradigms can lead to delayed but intact conditioning, rather than impaired performance in aged animals is also discussed. Overall, the eyeblink conditioning paradigm, when optimized for the age of the animal in the study, is an elegantly simple technique for assessment of associative learning and memory. When design caveats described above are taken into account, this important type of memory, with its well-defined neural substrates, should definitely be included in cognitive assessment batteries for the aged.

## Introduction

Eyeblink conditioning, a prototypical form of classical conditioning, is an elegantly simple but effective test of associative learning capacity. The use of the eyeblink as a means to explore neural function appeared at the end of the nineteenth century (Zwaardemaker and Lans, 1899), and gained momentum early in the twentieth century as used by Cason, Dodge, Hilgard, and Switzer (Cason, 1922; Dodge, 1927; Switzer, 1930; Hilgard, 1931). Ernest Hilgard used a comparative approach to describe this form of learning in a series of studies conducted using dogs, monkeys, and humans, and provided evidence of a common central mechanism in associative learning across these species (Hilgard, 1931; Hilgard and Marquis, 1935, 1936a,b; Marquis and Hilgard, 1936, 1937). Its utility as a method to identify age-related decrements in associative learning ability surfaced several years later (Gakkel and Zinina, 1953). Specific age-related changes in associative learning ability became more apparent when Braun and Geiselhart (1959) discovered that old adults between 62 and 84 years of age displayed significantly poorer associative learning than did children and young adults. Thus, eyeblink conditioning has been a powerful tool for revealing distinct age-related changes in the neural systems responsible for this behavior across a wide variety of mammals. The changes that occur in performance on eyeblink conditioning paradigms with increasing age largely have been attributed to changes in the neural systems engaged during eyeblink conditioning paradigms. While this may in part be true, it is also possible that some age-related decrements in performance reflect changes in the sensitivity to the parameters of the eyeblink task itself, rather than to defects in the neural systems that underlie the learning of these associations. Despite its wide use in learning and memory research in the aged, a comprehensive cross-species review of the how task parameters in the eyeblink conditioning paradigm influence the outcome of these studies in the aged has not been compiled (for a review on the human literature, see Woodruff-Pak, 2000).

For this overview, the use of this method, and its application, and interpretation for experiments on associative memory during aging is organized as follows: (1) the two associative single-cue eyeblink conditioning paradigms most commonly used in the aged and across species are introduced; (2) the role that non-associative factors may play in performance differences across age is discussed; (3) the effect of specific task parameters on acquisition and extinction of the conditioned eyeblink response is discussed; (4) how the task parameters discussed in #3 above may affect aging studies in humans, rabbits, and rodents is discussed; and finally, (5) the need to develop a set of procedural references with which to optimize the parameters for future eyeblink conditioning studies is emphasized.

## Associative eyeblink conditioning paradigms and behavioral studies of associative memory

**Figure 1:**
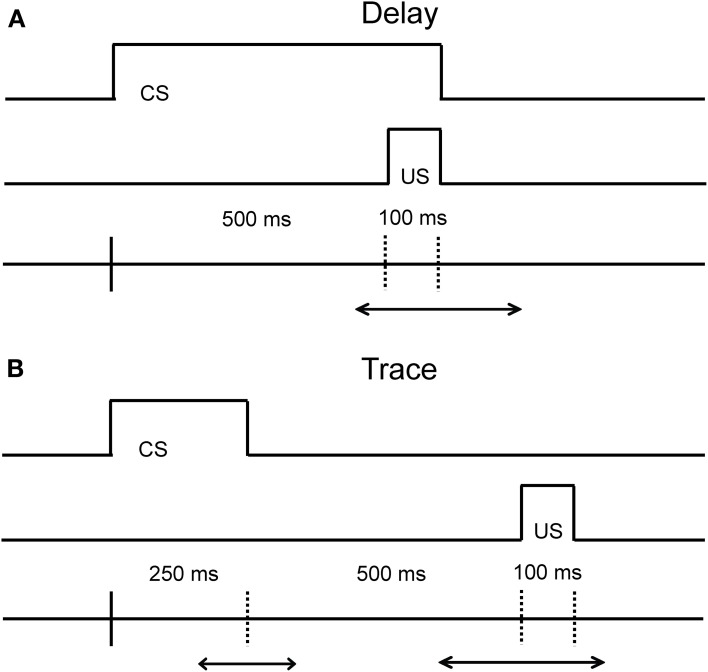
**Diagram of the temporal relationship in the Delay (A) and Trace (B) eyeblink conditioning paradigms.** The main difference between the delay and trace conditioning is that the CS and US do not overlap in the trace conditioning paradigm. The duration of the CS in the delay paradigm can vary in time. The duration of the CS and the trace interval in the trace paradigm can also vary in time. The optimal duration of the CS and the trace interval in the delay and trace paradigm is species-specific.

Eyeblink conditioning is a behavioral associative learning paradigm that pairs a neutral conditioned stimulus (CS; e.g., either auditory or visual stimuli) with a salient, normally aversive, unconditioned stimulus (US; e.g., an airpuff to the eye or a slight electric shock the eyelid) that elicits an eyeblink. After several pairings, the CS becomes predictive of the US, and ultimately leads to the acquisition of a conditioned response (CR; e.g., an eyeblink) that precedes the US. The temporal relationship between the time that the CS is presented and the US is delivered is critical both with respect to the underlying neural circuits engaged, and with respect to the acquisition rate of learning (Figure 1 illustrates the differences between these task variants). Both the delay and trace conditioning paradigms have played a critical role in our current understanding of the different neural systems that are related to associative learning.

Our current understanding of the neural systems that mediate associative learning come from numerous studies in the rabbit, rat, and mouse. As is evident from Figure 1, the critical difference between the delay and trace conditioning paradigms resides in the timing and duration between the onset and offset of the CS and US. In the delay conditioning paradigm, the onset of the CS precedes the onset of the US by a short or long duration, and the offset of the CS and US co-terminate. Acquisition of the CR in this paradigm is dependent upon the interpositus nucleus of the cerebellum, as a lesion of this structure abolishes the ability to learn the relationship between the CS and US in rabbits and rats (Thompson, 1986; Skelton, 1988; Krupa and Thompson, 1997; Woodruff-Pak et al., 2010). For the trace conditioning paradigm, the CS and US are separated by a stimulus-free interval leaving a temporal gap to be bridged for this version of the task. Acquisition of the CR in the trace conditioning paradigm appears to be also reliant upon the cerebellum (Woodruff-Pak et al., 1985), but the hippocampus and medial prefrontal regions (mPFC) are also critical for optimal task performance as lesions in these regions lead to poorer task acquisition and task retention (Solomon et al., 1986; Moyer et al., 1990; Kim et al., 1995; Tseng et al., 2004). For example, aspiration lesions of the hippocampus in rabbits disrupts the acquisition of new trace associations, but leaves previously acquired trace associations intact (Kim et al., 1995). Additionally, areas in prefrontal cortex have been found to contribute to and play an important role in trace conditioning (for an extensive review of the contribution of prefrontal cortex on trace eyeblink conditioning, see Weiss and Disterhoft, 2011). For example, aspiration lesions of the caudal area of the medial prefrontal cortex (mPFC) in rabbits also disrupt the acquisition of the CR in the trace conditioning paradigm (Kronforst-Collins and Disterhoft, 1998), whereas aspiration lesions of the rostral area of mPFC produce extinction impairments following trace conditioning (Weible et al., 2000). Lesion studies that impair the acquisition, extinction, or retention of CRs are important to the interpretation of aging studies as they can facilitate the development of hypotheses concerning the localization of underlying changes in the neural substrates responsible for associative learning. One major challenge of the use of associative learning paradigms in the aged is the necessity to tease apart the influence of non-associative factors on this type of learning. The majority of the eyeblink conditioning studies to date in the aged have been conducted in humans, rabbits, and rodents. Therefore, this review will focus on non-associative factors that pertain to the specific CS modality used in these tasks, as well as how adjustments to these task parameters affect acquisition performance within and between these species as a function of age.

## Non-associative factors as a source of variance in associative learning paradigms

Aging is accompanied by numerous changes that impact both higher-level cognitive functions and lower-level sensory functions. Degradation of the visual, auditory, somatosensory, gustatory, and olfactory systems have all been documented in the aged (e.g., Deems and Doty, 1987; Doty, 1989; Ohlemiller, 2004, 2006, 2008, 2009; McGinty and Truscott, 2006; Low Choy et al., 2007; Michael and Bron, 2011). Considering how these non-associative changes may impact the results of any eyeblink conditioning study in the aged is extremely important to avoid potential unforeseen confounds (Powell et al., 1991). Traditionally, age-related changes of the sensory systems have been attributed to deteriorated peripheral apparatus, which ultimately impacts the acuity of that sensory system. For example, hardening and yellowing of the lens has been found to contribute to poorer visual acuity (McGinty and Truscott, 2006; Michael and Bron, 2011), while a loss of outer hair cells and spiral ganglion cells has been shown to contribute to age-related hearing loss (Ohlemiller, 2004). To date the majority of eyeblink conditioning studies in the aged have used either a visual or auditory stimulus as the CS, and the acuity of the these sensory systems are rarely or insufficiently screened in the aged before training (Solomon et al., 1989, 1991; Thompson et al., 1996; Woodruff-Pak and Jaeger, 1998).

Additionally, changes in processing speed have been well-documented in the aged (Salthouse, 1996; Carp et al., 2010), and need to be accounted for in studies that examine the precise reaction time of an eyeblink CR. Directly measuring eyeblink reaction time is an analytical approach that can easily be implemented in studies of human participants to control for this. Woodruff-Pak and Jaeger (1998) found that eyeblink reaction time, after removing skewed data by restricting and analyzing participant average reaction times that were less than 500 ms, does not change with age. Measuring eyeblink reaction time in non-human animals is more challenging. One strategy that has been applied in aging studies is to examine both hippocampal-dependent and cerebellar-dependent learning in the same study to control for non-associative factors, such as sensory degradation and slower reaction time. The rationale behind this strategy is that both hippocampal and cerebellar circuits would be equally impacted by degraded peripheral apparatus, and the normal acquisition of learned CRs in either the trace or the delay eyeblink conditioning paradigm could, in principle, rule out a non-associative effect on associative learning. While this strategy may control for some changes in an altered peripheral apparatus and processing speed of the response, age-related changes in sensory systems are not necessarily isolated to the periphery. Changes in central processing of sensory information also have been found in both the visual and auditory systems of older people and other animals (Schmolesky et al., 2000; Dietrich et al., 2001; Yu et al., 2006; Yang et al., 2008; Zhang et al., 2008; Juarez-Salinas et al., 2010; Recanzone et al., 2011). This adds an additional layer of complexity for the design of eyeblink conditioning experiments for the aged, as both peripheral and central sensory processing impairments may contribute to decrements in performance of associative learning.

## The conditioned stimulus: back to the basics

The composition of the CS itself is one of the fundamental task parameters of any eyeblink conditioning paradigm. The most important aspect of the CS is that it must be neutral, which implies that it does not elicit the desired response before it is associated with the US. Selecting an appropriate CS is critical to all studies of associative learning in the aged, especially in animals that experience concomitant age-related sensory decline. As mentioned previously, the visual and auditory modalities have been used primarily as the CS in eyeblink conditioning in young and aging subjects (Hilgard, 1931; Hilgard and Marquis, 1936b; Braun and Geiselhart, 1959; Gormezano et al., 1962; Schneiderman and Gormezano, 1964; Woodruff-Pak et al., 1985; Finkbiner and Woodruff-Pak, 1991; Weiss and Thompson, 1991; Clark and Zola, 1998), although both olfactory and somatosensory stimuli have been succesfully used to evoke associative learning in young and aged subjects (Moore and Murphy, 1999; Galvez et al., 2011; Farley et al., 2011). Because abrupt high intensity visual and auditory stimuli can evoke startle responses that can manifest as eyeblink responses (Lang and Davis, 2006), examining the startle sensitivity of the subject along with pseudoconditioning have been suggested as good controls for this potential confound (Powell et al., 1991; Thompson et al., 1996).

**Figure 2:**
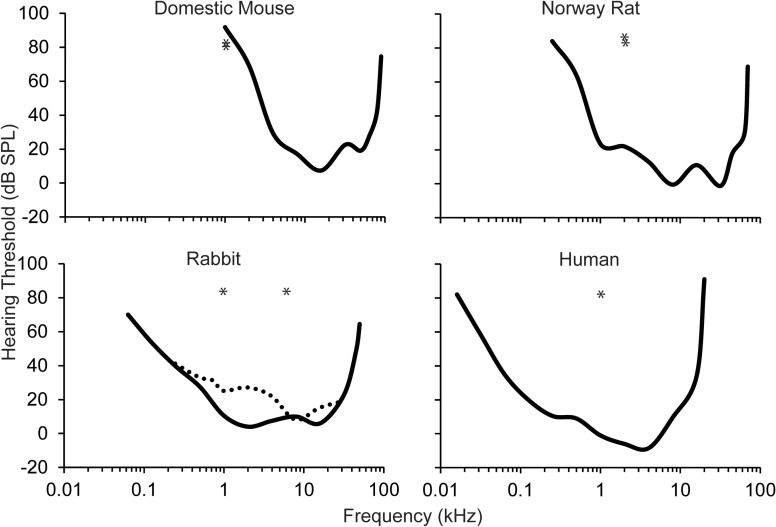
**Behavioral audiograms (solid lines) for the domestic mouse, Norway rat, rabbit, and human.** Audigram based upon eyeblink conditioning (dashed line) in the rabbit. The frequency of the auditory CS used across the majority of eyeblink conditioning studies is marked (asterisks). However, the importance of optimizing the auditory stimulus for each species is highlighted in studies in mice where the CS that has been used is near the bottom of the audible range for this species. Audiograms derived from: the domestic mouse (Koay et al., 2002), the Norway rat (Heffner et al., 1994), the rabbit (Heffner and Masterton, 1980; Martin et al., 1980), and the human (Kojima, 1990; Jackson et al., 1999).

The auditory stimuli have ranged from simple pure tones to white noise. The most common auditory stimulus used in eyeblink conditioning studies is a 1 kHz low frequency pure tone. Unfortunately, this auditory stimulus is generally 1–2 octaves below the best audible frequency of the animal's behavioral audiogram within the study (see Figure 2). In humans, the best audible frequency is approximately 4 kHz (Kojima, 1990). Domestic rabbits have a slightly lower best audible frequency at 2–8 kHz (Heffner and Masterton, 1980; Martin et al., 1980). Numerous studies of the rat and mouse have also used the 1 kHz auditory stimulus as the CS (Kishimoto et al., 2001a,b,c,d; Woodruff-Pak et al., 2010). However, the best audible frequency in rats and mice falls between 8 and 32 kHz (Heffner et al., 1994, 2001, 2006; Heffner and Heffner, 2007). Recent eyeblink conditioning paradigms have used mice due to their short lifespan and the ability to create transgenic strains. Unfortunately, because the lower limit of the audible range in mice is approximately 1 kHz, the validity of any result found in studies that use the 1 kHz pure tone CS in mice needs to be questioned, especially when examining age-related changes. Mild or severe changes in peripheral sensitivity with age will dramatically change the ability of the mouse to use this stimulus as an effective CS, especially since it is at the lower end of the audible range.

Furthermore, sensory acuity must be addressed in future eyeblink conditioning studies as a potential non-associative contributing factor in aging studies because hearing loss is both a peripheral and central phenomenon. For auditory stimuli, age-related hearing loss may confound task performance, because CRs to auditory stimuli are intensity-dependent (Walker, 1960; Scavio and Gormezano, 1974). The use of non-optimal CS frequencies may bias the results of eyeblink conditioning studies against the possibility of good performance in the aged. Only a few studies have tested the sensory acuity of the sensory system that is conveying the sensory signal from the peripheral apparatus to the brain of their participants (Solomon et al., 1989, 1991, 1995). These potential problems can obviously be resolved in future studies by assessing auditory function directly, using the animal's best audible frequency in the test, and by normalizing a young control group to the same perceptual level of the auditory stimulus that is observed in aged populations. These recommendations are for aged populations with changes in peripheral spectral processing deficits, and not for those with central changes that are commonly associated with temporal processing deficits related to poor speech perception (see Walton, 2010).

If the acquisition of the CR is truly independent of the sensory modality of the CS in eyeblink conditioning studies, then the acquisition rate of CRs might be expected to be similar across modalities. Consistent with this idea, a recent study (Steinmetz et al., 2009) matched the perceptual intensity of auditory and visual stimuli in young humans to determine if the modality of the CS influenced the outcome in the acquisition rate of the CR. Interestingly, no differences were detected in the overall rate of acquisition of the CRs to perceptually matched auditory and visual stimuli. Additionally, the first age-related study to demonstrate impaired acquisition of conditioned eyeblink responses in humans used a visual stimulus as the CS (Kimble and Pennypacker, 1963). Lastly, the use of other sensory modalities is recommended in aged participants with degraded visual abilities. In a recent study, Galvez et al. (2011) used both somatosensory and auditory CSs in aged C57B16 mice, and found decrements in associative learning as a function of sensory modality and age, with deficits emerging earlier with the auditory CS. The utilization of stimuli across two sensory modalities to serve as the CS in eyeblink conditioning studies in the aged may reconcile and tease apart sensory processing deficits from cognitive associative deficits in populations with degraded sensory sensitivity.

## The interstimulus-interval impacts associative learning in the aged

One of the many advantages of the eyeblink conditioning procedure resides in the robustness of this research design to engage the known neural substrates of associative learning and memory in animals over the lifespan. Because the test can be administered without requiring language, and the same neural circuits are engaged in all species tested (see above), eyeblink conditioning has been an outstandingly versatile method to explore the biological basis of cognitive aging. The primary task parameter shown to engage different neural systems of associative learning is the interstimulus interval (ISI) between the CS and US. The engagement of the cerebellum and its associated nuclei, and or the hippocampus is critically dependent upon the temporal relationship between the CS and the US. However, the ISI necessary for optimal conditioning has been shown to be species-specific and age-dependent. For example, an ISI greater than 1000 ms is required for trace conditioning to engage the hippocampus in humans (Clark and Squire, 1998), while an ISI of ~300 ms can engage the hippocampus in smaller animals, such as rodents and rabbits (Moyer et al., 1990; Brown et al., 2010).

Numerous studies have shown age-related changes in associative learning in the delay and trace eyeblink conditioning paradigm (see Tables 1,2). In the delay paradigm, older human subjects are impaired at a 400 ms delay in a delay task compared with younger subjects (Woodruff-Pak and Thompson, 1988; Solomon et al., 1989; Woodruff-Pak and Jaeger, 1998). This result suggests that, at this interval, cerebellar circuits in older persons during delay conditioning are disadvantaged in some way. Because Solomon et al. (1991) has shown that the age-related impairment observed at the 400 ms interval in the delay paradigm is attenuated when longer ISIs of 650 and 900 ms are used between the CS and US, it appears that cerebellar circuits are still capable of forming new associations in the aged, but other, probably central factors contribute to the observed impaired performance in the aged. It is interesting to speculate that there may be altered temporal dynamics in the cerebellum, possibly related to general speed of processing or temporal integration defects that have been noted in the elderly that may contribute to the age-related change in optimal interval for conditioning to occur. Because these results suggest that the ISI can be optimized in aged populations to reveal associative learning capacity, utilization of carefully chosen task parameters for the aged is particularly critical for an unbiased examination of age-related alterations in systems linked to associative learning and memory.

**Table 1:** **Parameters used in the delay conditioning paradigms in young humans, rabbits, and rodents**.

**Study**	**Age sensitive**	**CS duration (ms)**	**Stimulus type**	**US duration (ms)**	**Trials to Criterion/Asymptote**	**CR (%)**
**HUMAN**						
Braun and Geiselhart, 1959	Yes	1000	Light	500	60	60
Kimble and Pennypacker, 1963	Yes	500	Light	50	35	50
Solomon et al., 1989	Yes	500	Tone (1 kHz)	100	30	80
Solomon et al., 1991	Yes	400	Tone (1 kHz)	100	30	70
	No	750	Tone (1 kHz)	100	40	70
	No	1000	Tone (1 kHz)	100	20	65
**RABBIT**						
Powell et al., 1984	Yes	750	Tone (1.2 kHz)	250	192	70
Graves and Solomon, 1985	No	500	Tone (1.2 kHz)	50	300	90
Solomon et al., 1995	Yes	500	Tone (1 kHz)	100	900	70
Rose et al., 2007	Yes	750	Tone (1 kHz)	100	810	75
Woodruff-Pak et al., 2007	Yes	750	Tone (1 kHz)	100	630	25
		200	Tone (1 kHz)	100	1500	80
**RODENTS**						
Paredes et al., 2009	Yes	400	Tone (3 kHz)	100	250	70
Woodruff-Pak et al., 2010	Yes	600	Tone (1 kHz)	100	180	70

**Table 2:** **Parameters used in the trace conditioning paradigms in young humans, rabbits, and rodents**.

**Study**	**Age sensitive**	**CS duration (ms)**	**Stimulus type**	**Trace interval (ms)**	**US duration (ms)**	**Trials to Criterion/Asymptote**	**CR (%)**
**HUMAN**							
Finkbiner and Woodruff-Pak, 1991		400	Tone (1 kHz)	0	100	30	80
		400	Tone (1 kHz)	500	100	20	63
		400	Tone (1 kHz)	800	100	41	46
		400	Tone (1 kHz)	1100	100	66	47
	Yes	400	Tone (1 kHz)	1400	100	64	47
		400	Tone (1 kHz)	1700	100	101	29
**RABBIT**							
Graves and Solomon, 1985	Yes	450	Tone (1.2 kHz)	500	50	700	75
Woodruff-Pak et al., 1987	Yes	250	Tone	500	100	378	89
Moyer et al., 2000	Yes	100	Tone (6 kHz)	500	150	733	80
Rose et al., 2007		200	Tone (1 kHz)	300	100	810	75
		200	Tone (1 kHz)	400	100	720	25
		200	Tone (1 kHz)	500	100	1500	20
**RODENTS**							
Matthews et al., 2009	No	250	Tone	250	50	150	80
Weiss et al., 1999		250	Tone (8 kHz)	250	100	350	*70
		250	Tone (8 kHz)	500	100	500	*20

* Reported as percent late CR.

## Age-related changes in eyeblink conditioning

A significant understanding of age-related impairments in associative learning and memory has come from eyeblink conditioning studies in humans, rabbits, and rodents (Kennard and Woodruff-Pak, 2011). A large body of research in humans suggests that the sensitivity of humans for eyeblink conditioning follows an inverted U-shape function over the lifespan (Woodruff and Steinmetz, 2000). The majority of eyeblink conditioning studies suggest that, in adulthood, associative learning is inversely correlated with age. Significant impairments in eyeblink conditioning typically begin to manifest themselves at 40 and 50 years of age (Woodruff-Pak and Thompson, 1988). Responses from men and women are often pooled together in eyeblink conditioning studies due to unbalanced designs (Cheng et al., 2010), but a lack of significant gender differences have been reported (Finkbiner and Woodruff-Pak, 1991). Age-related changes in performance on eyeblink conditioning paradigms have been attributed to changes in the neural systems that are engaged during each paradigm. Much of our current knowledge of the changes in the neural systems responsible for acquisition and retention of CRs come from studies in rodents and rabbits.

The rodent model of aging has become increasingly widely used for understanding mammalian aging for a number of reasons. Among these is the fact that similar brain and behavioral changes have been observed in these animals across age as is also observed in humans. Additionally, both the rat and the mouse have a relatively short lifespan, are easy to breed, relatively easy to assess behaviorally, and in particular, mice are easily used to generate transgenic models (Vogel et al., 2002; Tseng et al., 2004; Kennard and Woodruff-Pak, 2011). Both the rat and the mouse display impaired acquisition performance in eyeblink conditioning paradigms with age when compared to younger mature animals (Weiss and Thompson, 1991, 1992; Kishimoto et al., 2001a; Knuttinen et al., 2001; Vogel et al., 2002; Woodruff-Pak et al., 2006, 2010). Unfortunately, lifespan differences that impact performance within species and between different strains make it difficult to standardize the parameter space of eyeblink conditioning paradigms in rodents. Therefore, when designing eyeblink conditioning experiments in rodents, it is important to understand how the parameters chosen may interact with the specific rodent used in the study.

The duration of the CS has little impact on the rate of acquisition of the CR in the delay conditioning paradigm in rodents. Age, on the other hand, greatly impacts the rate of acquisition performance. For example, Weiss and Thompson (1991) found that their young and adult Fischer-344 (F-344) rats displayed approximately 60% CRs on the third day of training, while at this time point during acquisition, middle-aged 18 month and aged 30 month F-344 rats showed 40% CRs. One variable that does need to be taken into account when looking at different rodents is that there can be large variations in the actual lifespan of a given rat strain, as well as differences in performance levels even at young ages between strains. This is illustrated in a study that used the F344 × BN F1 hybrid strain that has a longer life expectancy and even at young ages shows 80% correct CRs at a time when F344s would show 60% CRs (Weiss and Thompson, 1992). This study also highlighted the importance of carrying training sessions out longer when conducting experiments that compare young and old rats. By extending the number of training trial sessions, Weiss and Thompson found that cerebellar-dependent associative learning is slower to appear, but that old rats will eventually learn, given more trials. Thus, by restricting the number trial sessions to match young animal performance can potentially bias our understanding of associative learning in aged rats. This observation lends further support to a speed of processing and temporal integration defect interpretation of associative learning impairment in aging.

The trace conditioning paradigm appears to be more sensitive than the delay condition paradigm at revealing age-related associative learning impairments in rodents. This is presumably due to the contribution of altered hippocampal function to the deficit observed in the aged animals. Knuttinen et al. (2001) found that under the conditions of their experiment, senescent F344 × BN F1 hybrid rats were slower to learn in a delay conditioning paradigm, but were impaired on the trace conditioning paradigm when it was administered. This result suggests that hippocampal-dependent associative learning is age-sensitive, and that it is a method that can be combined with other age-sensitive tests to form a comprehensive battery to assess cognitive aging (Knuttinen et al., 2001).

## Discussion

Age-related impairments in associative learning have been reported in humans, rabbits, rats, and mice, and compelling evidence has accumulated over the years suggesting that the changes in the neural systems responsible for this type of associative learning are the source of this impairment. The degree of performance differences between younger and older populations can be dramatically reduced, however, when optimal task parameters are identified for older subjects, and age-sensitive tests are applied.

For example, because increasing the ISI can attenuate poorer performance in aged humans, this suggests that associative learning is not abolished with age. However, the role that various non-associative factors play in the observed performance differences in aging has not been completely evaluated. Using optimal task parameters is the necessary first step that will help tease apart the role of non-associative factors on poor task performance in the aged. These will include accounting for both age-related peripheral and central processing deficits and how these factors may contribute to poorer task performance in eyeblink conditioning paradigms. In the former case, this can be accomplished by screening and controlling for age-related changes in sensory acuity. If changes are found peripherally (changes in auditory or visual acuity), then application of appropriate controls that adjust and match the sensory acuity across age groups should normalize task performance. One way to do this is to attenuate sensory acuity of young participants to match that of their aged counterparts. One potential downside of increasing the intensity of the stimulus for older adults (an alternate strategy) is that in some cases this may oversaturate sensory information and generate reflexive responses. The use of pure tones at the best audible frequency or broadband noise may mitigate some of the attenuation confounds that accompanies age-related hearing loss. In cases where age-related peripheral hearing loss is severe, the utilization of a visual, olfactory or somatosensory CS can be implemented, if the stimulus properties are easier to match between the young and elderly participants in a given study. This strategy was recently utilized by Galvez et al. (2011) who trained young and old C57/Bl6 mice with auditory and somatosensory stimuli during delay and trace conditioning. Acquisition performance varied as a function of conditioning paradigm, stimulus modality, and age. Importantly, the percentage of CRs to the vibration stimuli used on the vibrasse was greater than the auditory stimulus, and fell off at slower rate with age for both delay and trace conditioning paradigms. This study highlights how the optimization of the parameters in a species and age-specific manner can be used to elucidate and minimize the impact of non-associative factors in eyeblink conditioning paradigms to reveal real associative memory impairments.

On the other hand, even if peripheral sensory input is controlled, it is possible that there is a fundamental change in how the aged brain processes information. This may, however, be a true age effect that will be important to describe and understand. For example, age-related changes in cortical processing of lower level sensory information have been found in both the auditory and visual cortex (Schmolesky et al., 2000; Yu et al., 2006; Yang et al., 2008; Zhang et al., 2008; Juarez-Salinas et al., 2010; Recanzone et al., 2011). Changes in both rate and temporal response properties of auditory and visual cortical neurons suggest a fundamental change in how simple processing of sensory information propagates to higher level cognitive areas that are engaged during associative learning. Furthermore, age-related changes in the strength of hippocampal gamma oscillations have been reported *in vitro* (Vreugdenhil and Toescu, 2005; Lu et al., 2011). The optimization of the parameters in eyeblink conditioning paradigms for the aged is necessary to gain a better understanding of the fundamental neural changes that influence eyeblink conditioning in aged populations, and is necessary to facilitate therapeutic and behavioral remedial strategies aimed at attenuating decrements in associative learning and memory.

### Conflict of interest statement

The authors declare that the research was conducted in the absence of any commercial or financial relationships that could be construed as a potential conflict of interest.
